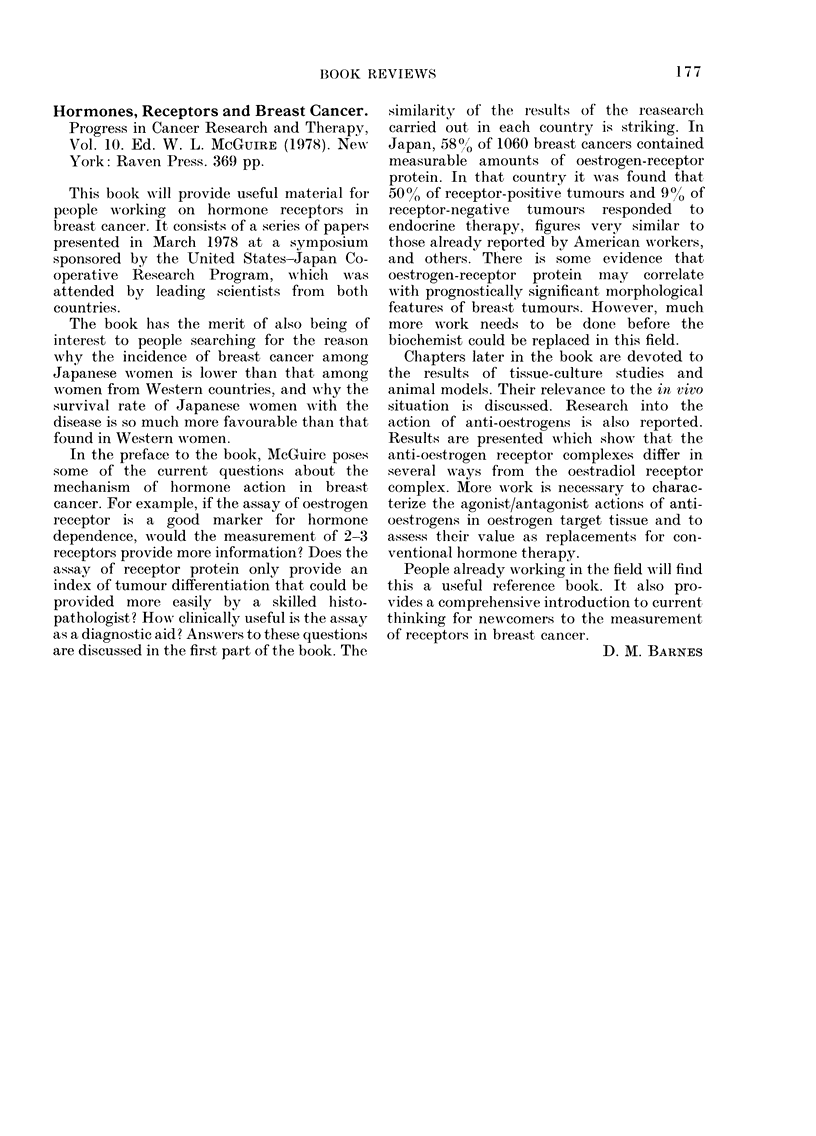# Hormones, Receptors and Breast Cancer

**Published:** 1979-07

**Authors:** D. M. Barnes


**Hormones, Receptors and Breast Cancer.** Progress in Cancer Research and Therapy, Vol. 10. Ed. W. L. McGuire (1978). New York: Raven Press. 369 pp.

This book will provide useful material for people working on hormone receptors in breast cancer. It consists of a series of papers presented in March 1978 at a symposium sponsored by the United States–Japan Co-operative Research Program, which was attended by leading scientists from both countries.

The book has the merit of also being of interest to people searching for the reason why the incidence of breast cancer among Japanese women is lower than that among women from Western countries, and why the survival rate of Japanese women with the disease is so much more favourable than that found in Western women.

In the preface to the book, McGuire poses some of the current questions about the mechanism of hormone action in breast cancer. For example, if the assay of oestrogen receptor is a good marker for hormone dependence, would the measurement of 2–3 receptors provide more information? Does the assay of receptor protein only provide an index of tumour differentiation that could be provided more easily by a skilled histopathologist? How clinically useful is the assay as a diagnostic aid? Answers to these questions are discussed in the first part of the book. The similarity of the results of the reasearch carried out in each country is striking. In Japan, 58% of 1060 breast cancers contained measurable amounts of oestrogen-receptor protein. In that country it was found that 50% of receptor-positive tumours and 9% of receptor-negative tumours responded to endocrine therapy, figures very similar to those already reported by American workers, and others. There is some evidence that oestrogen-receptor protein may correlate with prognostically significant morphological features of breast tumours. However, much more work needs to be done before the biochemist could be replaced in this field.

Chapters later in the book are devoted to the results of tissue-culture studies and animal models. Their relevance to the *in vivo* situation is discussed. Research into the action of anti-oestrogens is also reported. Results are presented which show that the anti-oestrogen receptor complexes differ in several ways from the oestradiol receptor complex. More work is necessary to characterize the agonist/antagonist actions of anti-oestrogens in oestrogen target tissue and to assess their value as replacements for conventional hormone therapy.

People already working in the field will find this a useful reference book. It also provides a comprehensive introduction to current thinking for newcomers to the measurement of receptors in breast cancer.

D. M. Barnes